# Is forest fecundity resistant to drought? Results from an 18‐yr rainfall‐reduction experiment

**DOI:** 10.1111/nph.16597

**Published:** 2020-05-02

**Authors:** Michał Bogdziewicz, Marcos Fernández‐Martínez, Josep M. Espelta, Romà Ogaya, Josep Penuelas

**Affiliations:** ^1^ Department of Systematic Zoology Faculty of Biology Adam Mickiewicz University 61‐614 Poznań Poland; ^2^ CREAF Cerdanyola del Vallès 08193 Catalonia Spain; ^3^ PLECO (Plants and Ecosystems) Department of Biology University of Antwerp 2610 Wilrijk Belgium; ^4^ Global Ecology Unit CSIC Cerdanyola del Vallès 08193 Catalonia Spain

**Keywords:** drought, fruit production, global change, mast seeding, rainfall reduction, reproduction, tree fecundity

## Abstract

- Recruitment is a primary determinant of the long‐term dynamics of plant populations in changing environments. However, little information is known about the effects of anthropogenic environmental changes on reproductive ecology of trees.
- We evaluated the impact of experimentally induced 18 yr of drought on reproduction of three contrasting forest trees: *Quercus ilex*, *Phillyrea latifolia* and *Arbutus unedo*.
- Rainfall reduction did not decrease tree fecundity. Drought, however, affected the allocation of resources in *Q. ilex* and *A. unedo* but not the more drought tolerant *P. latifolia*. Larger crop production by *Q. ilex* and *A. unedo* was associated with a stronger decrease in growth in the rainfall‐reduction plots compared with the control plots, suggesting that these species were able to maintain their fecundity by shifting their allocation of resources away from growth.
- Our results indicated resistance to change in tree fecundity in Mediterranean‐type forest subjected to an average 15% decrease in the amount of soil moisture, suggesting that these ecosystems may adapt to a progressive increase in arid conditions. However, the species‐specific reductions in growth may indirectly affect future fecundity and ultimately shift community composition, even without immediate direct effects of drought on tree fecundity.

## Introduction

Anthropogenic environmental changes are exerting increasing pressure on forests worldwide (Gauthier *et al.*, 2015; Seidl *et al.*, 2017), and accumulating evidence indicates that climate change is causing dramatic forest diebacks (Allen *et al.*, 2010; Seidl *et al.*, 2017; Lloret & Kitzberger, 2018). The critical question now concerns what ecosystems will follow from these profound transformations. Few studies, however, have compared the impacts of environmental change on the reproductive ecology of trees with other effects such as growth, carbon sequestration, mortality, or phenology (Barbeta *et al.*, 2013; Hacket‐Pain *et al.*, 2016; Zohner *et al.*, 2018; Luo *et al.*, 2020). Ecosystem services, such as mitigating the risk of avalanches, carbon storage, habitat availability and value for the economy and recreation, can suffer if reduced reproduction slows forest expansion or limits the recruitment of merchantable tree species and seed producers that support wildlife (McShea, 2000; Ostfeld & Keesing, 2000; Clark *et al.*, 2007, 2019; Bogdziewicz *et al.*, 2016). The volatility of seed production and our poor understanding of the mechanisms that govern it are challenges for anticipating alternations in forest reproduction (Bogdziewicz *et al.*, 2020a, 2020). Reliable predictive models are consequently not available, and the unpredictable recruitment of trees has become a key obstacle to understanding forest change (Ibáñez *et al.*, 2009; Zhu *et al.*, 2012).

Tree reproduction is sensitive to climate change (Mckone *et al.*, 1998; Pearse *et al.*, 2014; Monks *et al.*, 2016; Vacchiano *et al.*, 2017). Observational studies of long‐term trends in fecundity report both increases and decreases in mean reproductive effort in many important forest‐forming species (Richardson *et al.*, 2005; Mutke *et al.*, 2005; Redmond *et al.*, 2012; Allen *et al.*, 2014; Buechling *et al.*, 2016, Bogdziewicz *et al*., 2020b). A wide array of statistical tools used in these studies usually attributes these trends to global warming, but substantial uncertainty remains, as causality remains unestablished (Pesendorfer *et al.*, 2020). Experiments that simulate environmental conditions projected by models of global change are thus useful for predicting the impacts of environmental global change on the reproductive patterns of forest trees. Such experiments usually report substantial effects. For example, *Pinus taeda* growing in an atmosphere enriched in CO_2_ produced three‐fold as many cones as trees growing under natural conditions (LaDeau & Clark, 2001). Excluding rain reduced the production of seed biomass in *Quercus ilex* by 30% (Pérez‐Ramos *et al.*, 2010). Such experimental studies are nonetheless almost inevitably limited in time. A meta‐analysis of global‐change experiments have reported a dampening effect size of treatments (warming, nitrogen fertilisation, or drought) over time (Leuzinger *et al.*, 2011). Monitoring experimental systems as long as possible is thus desirable for assessing the long‐term impacts of global change on forest fecundity.

The drought experiment in the Prades Mountains in southern Catalonia has run since 1999, and is one of the longest running forest global‐change experiments in the world (Wu *et al.*, 2011; Barbeta *et al.*, 2013; Peñuelas *et al.*, 2018). The experiment is being conducted in a typical holm oak (*Q. ilex*) forest, where the oak is accompanied by other Mediterranean woody species with more (*Phillyrea latifolia*) or less (*Arbutus unedo*) drought tolerance (Peñuelas *et al.*, 2018). Important demographic effects have already been observed, such as a higher mortality of stems and reduced growth, especially in *Q. ilex* and *A. unedo* (Lloret *et al.*, 2004; Ogaya & Peñuelas, 2007b). The differences in the rates of growth and mortality between drought and control plots recorded at the beginning of the experiment eventually decreased after some years (Barbeta *et al.*, 2013; Liu *et al.*, 2015). These dampening effects were associated with decreased competition and high mortality after extreme drought, and possible morphological and physiological acclimation to drought during the study period may buffer forests against drier conditions (Barbeta *et al.*, 2013; Liu *et al.*, 2015; Peñuelas *et al.*, 2018).

We evaluated the impact of experimentally induced drought on the fecundity of the dominant forest trees at our experimental site: *Q. ilex*, *P. latifolia*, and *A. unedo*. All three species mast at our sites, that is reproduced by the spatiotemporally synchronous and temporally variable production of seeds (Kelly, 1994). The sensitivity of reproduction of mast‐seeding species to global change is predicted to be especially high due to hypersensitivity of masting plants seed production to variation in the weather (Mckone *et al.*, 1998; Monks *et al.*, 2016; Vacchiano *et al.*, 2017). In addition to the important trends in mean fecundity, changes in the strength of masting (that is the interannual variability and synchrony of reproduction) are crucial for tree fitness and forest regeneration, because masting is a life‐history trade‐off among missed reproductive opportunities in low‐seed years, increased pollination efficiency, and decreased seed predation in mast years (Kelly, 1994; Pearse *et al.*, 2016, Bogdziewicz *et al*., 2020b). We thus also tested the effects on coupling between plants and variation among years, in addition to evaluating the effects of drought on mean fruit production. We predicted that drought would reduce mean reproductive output in *Q. ilex* and *A. unedo*, but not *P. latifolia*, based on studies reporting that *P. latifolia* was much more drought tolerant than the other two species (prediction 1) (Ogaya & Peñuelas, 2007b; Barbeta *et al.*, 2013; Peñuelas *et al.*, 2018). We also predicted that the effect would dampen with time (prediction 2), paralleling the diminishing effects of drought on growth and mortality (Barbeta *et al.*, 2013). The theory of mast seeding predicts that more frequent adverse weather would increase the interannual variability of seed production and strengthen the synchrony of reproductive variation among trees (Rees *et al.*, 2002; Espelta *et al.*, 2008; Bogdziewicz *et al.*, 2018). We thus predicted an increase in annual variability and synchrony in all three species on droughted plots relative to control plots, but likely less so in drought‐resistant *P. latifolia* than in the other two species (prediction 3)*.* Finally, we expected that the drought experiment would induce variation in the strength of the trade‐off between growth and reproduction (prediction 4): the trade‐off would be stronger under stressful conditions (drought) (Martín *et al.*, 2015; Berdanier & Clark, 2016; Hacket‐Pain *et al.*, 2017).

## Materials and Methods

### Rainfall‐reduction experiment

We established the experimental site in 1999 on a 25% south‐facing slope in the Prades Holm oak forest in southern Catalonia (northeastern Spain) (41°21′N, 1°2′E; 930 m asl). As a result of former coppicing the forest has a very dense multistem canopy layer (15 433 stems ha^−1^) dominated by *Q. ilex* (5258 stems ha^−1^), *P. latifolia* (7675 stems ha^−1^), and *A. unedo* (1100 stems ha^‐1^), accompanied by other Mediterranean woody species that usually do not reach the upper canopy (e.g. *Erica arborea* and *Juniperus oxycedrus*) and occasional isolated deciduous trees (e.g. *Sorbus torminalis* and *Acer monspessulanum*). Holm oak forests in the Prades Mountains grow throughout the altitudinal range (400–1200 m), presenting closed canopies 3–10 m in height depending on site quality. This forest has been managed as a coppice for centuries, but has not been substantially disturbed for the last 70 yr.

The site has a Mediterranean climate with a mean annual temperature of 12.4°C and a mean annual precipitation of 610 mm during the study period (as described below in the Results section). Annual and seasonal precipitation are irregularly distributed, with annual precipitation ranging from 355 to 1021 mm in the 19 yr of this study. Spring and autumn are the wettest seasons, and summer drought usually lasts 3 months, during which time precipitation is *c. *10% of the annual total and coincides with the highest temperatures.

The experimental system consisted of eight 150‐m^2^ (15 × 10 m) plots delimited at the same altitude along the slope. We randomly selected half of the plots to receive the drought treatment, and the other half had natural conditions. We partially excluded precipitation in the drought treatment using PVC strips suspended 0.5–0.8 m above the soil and covering *c.* 30% of the plot surfaces, similar to conditions for other drought experiments in Mediterranean systems, and as projected by the IPPC panel for the region (Limousin *et al.*, 2008; IPCC, 2013). The precipitation was also excluded within a 2‐m wide buffer zone around the drought plots. Moreover, we dug a ditch 0.8 m in depth along the top edge (i.e. upslope) of the buffer zone of drought‐treatment plots to intercept water runoff.

We installed an automatic meteorological station (Campbell Scientific Inc., Logan, UT, USA) between the plots to monitor temperature, photosynthetic active radiation, air humidity, and precipitation, from which we obtained the Standardised Precipitation Evapotranspiration Index (SPEI) as a measure of atmospheric hydric conditions. SPEI is calculated as the difference between monthly precipitation and potential evapotranspiration (Beguería *et al.*, 2014). High and low SPEI values therefore indicated wet and drought conditions, respectively. We selected time scales of 3 (SPEI‐3) and 6 (SPEI‐6) months, because they fitted our annual data on plant growth and population dynamics analysed in earlier studies (Barbeta *et al.*, 2013; Liu *et al.*, 2015). The SPEI values are provided for month and time scale of calculation (e.g. SPEI_May3 refers to the water balance for March, April and May of a given year). We also measured soil moisture each month throughout the experiment by time‐domain reflectometry (TDR; Tektronix 1502C, Tektronix, Beaverton, OR, USA), connecting the time‐domain reflectometer to the ends of three stainless‐steel cylindrical rods, 25 cm long and fully driven into the soil, at four randomly selected locations per plot.

We randomly distributed 20 circular, waterproof baskets (27 cm in diameter with a 1.5‐mm mesh) on the ground in each of the eight plots and collected the fallen litter every 2 months from 1999 to 2017. The baskets were placed at least 2 m from the edge of the plot (4 m from the edge of the buffer zone). We placed a mesh wire at the top of the plots to trap litterfall inside baskets. Fruits were weighed after drying in an oven at 70°C to constant weight.

All living stems of all the species with a diameter of more than 2 cm at 50 cm height were tagged and their circumference was measured at 50 cm height with a metric tape. A line was painted on the exact point of the stem where circumference had been measured. Only one person was involved in the measurements to increase standardisation. We then calculated stem basal area increments (BAIs) and began in winter 2009 to also measure the individuals with diameters < 2 cm at the beginning of the study but which then attained or exceeded this size. Two cm is a standard cut‐off for such measurements used in Spanish forestry inventories. In total, we measured 735 stems in *Q. ilex*, 842 in *P. latifolia*, 145 in *A. unedo*. Average (SD) number of stems per plot equalled 105 (47) in *Q. ilex*, 128 (90) in *P. latifolia*, and 20 (14) in *A. unedo*.

### Focal species

Holm oak (*Quercus ilex* L.) is a drought‐tolerant tree that is widely distributed in the Mediterranean basin. Mock privet (*Phillyrea latifolia*) L. is a small tree associated with *Q. ilex* forests and more resistant to drought and high temperatures than *Q. ilex* (Ogaya & Peñuelas, 2003; Peñuelas *et al.*, 2018). Strawberry tree (*Arbutus unedo*) L. is another small tree typical of holm oak forests, less resistant to drought than *P. latifolia* (Ogaya & Peñuelas, 2003; Peñuelas *et al.*, 2018). The reproductive phenologies of *Q. ilex* and *P. latifolia* are typical for Mediterranean species. Flowering takes place in spring, fruit development in summer and fruit maturation and seed dispersal in autumn (Ogaya & Peñuelas, 2004). In *A. unedo*, flower bud formation occurs in the spring, but flowering takes place in the following autumn, and fruit development continues over a prolonged period until fruit matures in the autumn of the following year (Ogaya & Peñuelas, 2004).

### Statistical analysis

We evaluated the impact of reducing rain on soil‐water content by building a linear mixed model (LMM), with soil moisture as the response and treatment (control vs drought) as a fixed effect. Month and year were included as random intercepts.

We next evaluated the impact of excluding rain on fruit production (fruit dry mass per plot) in the model species using LMMs that included log‐transformed fruit mass as a response, with the interaction between treatment and year as fixed effects (predictions 1 and 2). The interaction was included to test for possible dampening effects of the drought treatment on fruit dry mass production. We built a separate model for each species. Each model also included plot as a random intercept and SPEI as a covariate. The specific month for each SPEI was preidentified for each species by fitting a partial least squares regression (PLS) of fruit production vs all possible SPEI values. PLS is designed to analyse a large array of related predictor variables, with insufficient sample sizes relative to the number of independent variables (Carrascal *et al.*, 2009). The number of plot‐years for the analysis was 144 for *Q. ilex* and *P. latifolia* and 126 for *A. unedo*, which was absent in one of the plots.

We next evaluated the influence of the experimental drought on the interannual variability and synchrony of fruit dry mass production per plot (prediction 2). We estimated the synchrony of fruit production for each treatment by calculating the mean pairwise cross‐correlation of fruit production over all plots of a treatment. The cross‐correlations were calculated using the mSynch function in the ncf package (Bjornstad & Falck, 2001). We also calculated measures of interannual variability for the treatments using the coefficient of variation (CV) and a proportional variability index (PV) (Heath, 2006). We used both indexes because CV can be skewed by its mean‐dependency, while PV is not (Fernández‐Martínez *et al.*, 2018). Yet, CV is widely used to measure interannual variation in seed production, allowing among‐studies comparisons. The corresponding 95% confidence intervals (CIs) for the focal values were calculated by bootstrap resampling with 1000 replications.

We built LMMs that included annual stem diameter increment (BAI) as a response to test whether excluding rain changes the trade‐off between growth and fruit dry mass production in our model species, with the interaction between log‐transformed fruit mass and treatment as a fixed effect (prediction 4). We built a separate model for each species. Each model included plot and tree as random intercepts. We also included SPEI values as covariates, which were similarly preselected for each species as in the models testing for the effects of treatment on fruiting. The BAIs were standardised within trees before inclusion in the models (i.e. we extracted the mean of each value and then divided it by the standard error). In each model, we also included a matrix for a natural cubic spline (*df* = 5) of tree size to account for growth‐related trends in BAI, using the *ns* function from the splines package. The sample sizes for these models, that is per stem per year observations, were 11 288 for *Q. ilex* (735 stems measured), 15 301 for *P. latifolia* (842 stems), and 2242 for *A. unedo* (145 stems).

We fitted all models using the glmmTMB package (Brooks *et al.*, 2017) in R. Models were validated, including tests for homoscedasticity and normality of residuals and potential outliers, using the dharma package (Hartig, 2017). We also explored all models with temporal autocorrelation structures (ar1) and retained or rejected them based on standard Akaike information criteria.

## Results

**Fig. 1:**
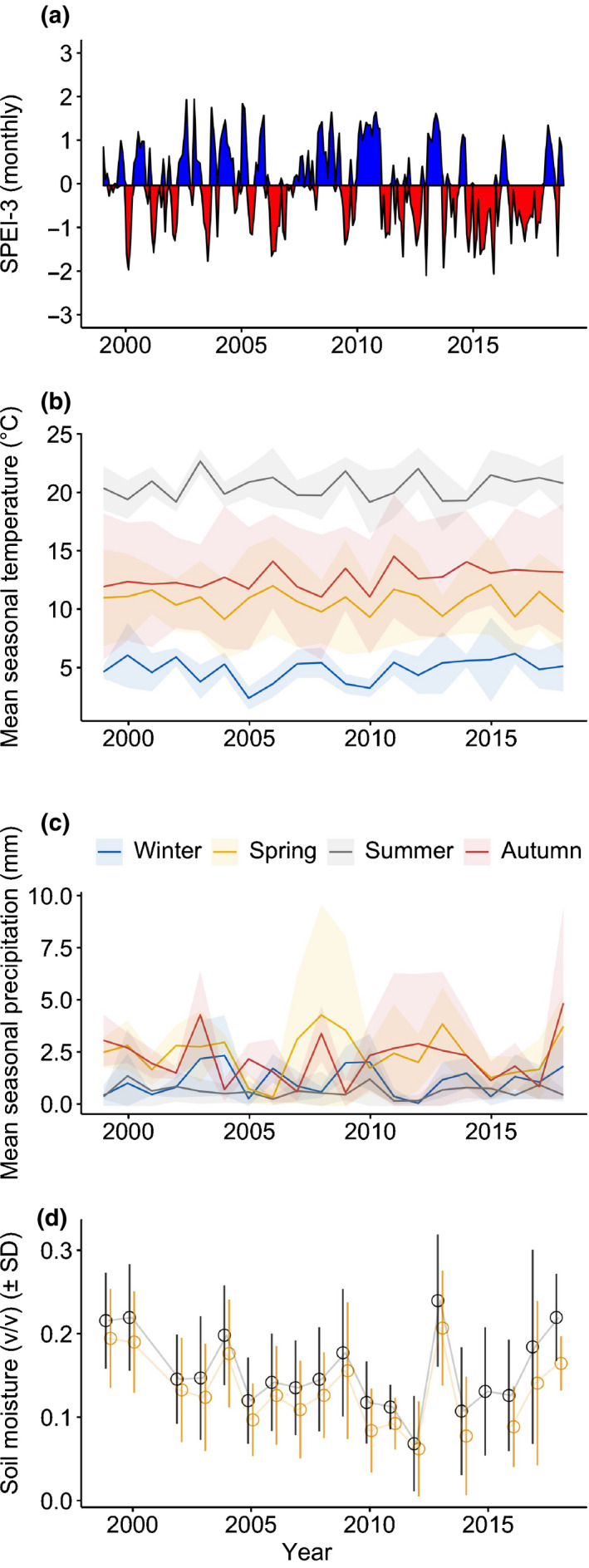
Abiotic conditions at the experimental site in the Prades Mountains in southern Catalonia. (a) SPEI‐3, (b) temperature, (c) precipitation and (d) soil moisture. Data for soil moisture were not collected in 2001 and 2015 due to equipment malfunction. Shading at (b) and (c) represent standard deviations of the means (calculated within years, across months). Black colour at (d) indicates the control, while yellow is rainfall‐reduction treatment; v/v is volumetric moisture content.

Drought treatment decreased the soil moisture by *c.* 10–30% during the study period (*z* = 8.32, *P* < 0.001), with larger differences during periods of rain (Fig. 1). Drought treatment decreased the amount of soil moisture by *c.* 20% or more during these periods but by < 10% during dry periods. Median reduction equalled 13%, while the mean was 15%.

**Fig. 2:**
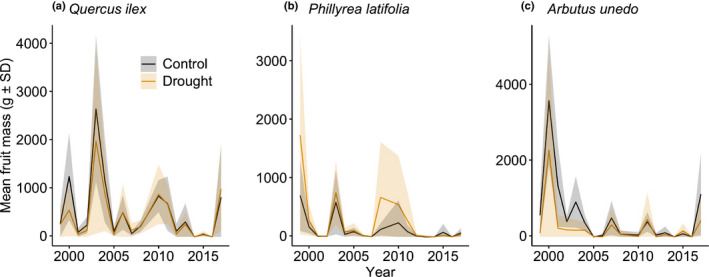
Fruit production by (a) *Quercus ilex*, (b) *Phillyrea latifolia* and (c) *Arbutus unedo* in the control and drought plots. The solid lines and shaded areas are annual means and associated SD, respectively. The number of observations of reproductive events is 144 (plot‐years) for *Q. ilex* and *P. latifolia* and 126 for *A. unedo*, which was absent in one of the plots. *Q. ilex* and *P. latifolia* were observed at eight plots, while *A. unedo* was observed at seven plots.

Drought treatment did not decrease fruit dry mass production in any of the species (Fig. 2), contrary to our expectations (prediction 1). The interaction between treatment and year was not significant for any of the species (*P* > 0.10) (prediction 2). Drought treatment without the interaction term did not significantly affect fruit production by *Q. ilex* (*z* = −0.64, *P* = 0.52), *P. latifolia* (*z* = 0.10, *P* = 0.92), or *A. unedo* (*z* = −1.60, *P* = 0.11). SPEI was positively correlated with fruit dry mass production per plot for all three species (*Q. ilex*: SPEI_March3, *z* = 4.49, *P *< 0.001, *P. latifolia*: SPEI_March3, *z* = 5.29, *P *< 0.001, *A. unedo*: SPEI_December3, *z* = 2.59, *P* = 0.01).

**Fig. 3:**
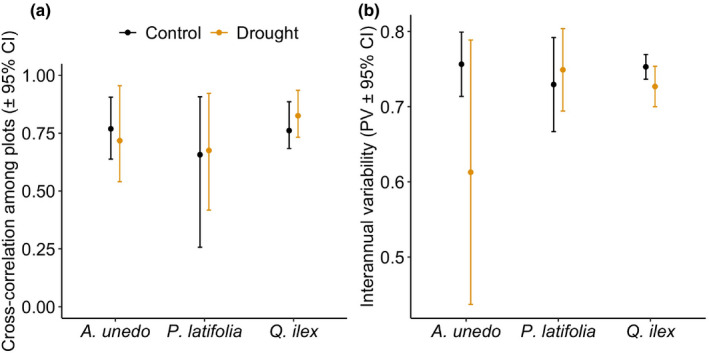
Synchrony (a) and interannual variability (PV) (b) of fruit production by *Quercus ilex*, *Phillyrea latifolia* and *Arbutus unedo* in the control and drought plots. Synchrony was measured by mean pairwise Pearson correlation among plots. Coefficient of variation not shown.

The variability of fruit dry mass production among years was high for all species, with positive pairwise cross‐correlations in fruiting among plots (mean cross‐correlation > 0.60 for all species, Fig. 3). Both interannual variability, as measured by either PV or CV, and synchrony, were unaffected by reducing rain for all three species (*P* > 0.05), contrary to our expectations (prediction 3).

**Fig. 4:**
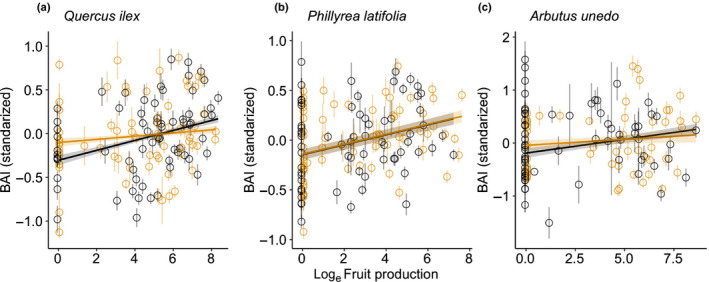
Scatterplots of standardised basal area increment (BAI) and fruit production for (a) *Quercus ilex*, (b) *Phillyrea latifolia*, and (c) *Arbutus unedo* in the control and drought plots. The lines and shaded areas are the linear mixed model predictions and associated 95% confidence intervals, respectively. Points and whiskers are plot‐year means and associated SE, respectively. Black represents the control, and yellow represents experimental rainfall exclusion. The sample sizes for these models are 11 288 for *Q. ilex* (735 stems measured), 15 301 for *P. latifolia* (842 stems), and 2242 for *A. unedo* (145 stems).

Reducing rain affected reproductive allocation for *Q. ilex* and *A. unedo* but not *P. latifolia* (Fig. 4) (prediction 4). *Quercus ilex* fruit production was positively correlated with growth in the control plots (β (SE) = 0.06 (0.005); *z* = 10.85, *P* < 0.001), but the slope of the relationship (β (SE) = 0.04 (0.005); *z* = 7.50, *P* < 0.001) was lower in the drought plots (interaction term: *z* = −5.57, *P* < 0.001). Fruit production by *A. unedo* was similarly positively correlated with growth in the control plots (β (SE) = 0.05 (0.009); *z* = 5.86, *P* < 0.001), but the slope of the relationship (β (SE) = 0.02 (0.01); *z* = 2.06, *P* = 0.04) was lower in the drought plots (interaction term: *z* = −2.15, *P* = 0.03). *Phillyrea latifolia* growth was positively correlated with reproduction (β (SE) = 0.05 (0.003); *z* = 14.78, *P* < 0.001), and the induced drought did not affect this pattern (interaction term: *z* = −0.97, *P* = 0.33). Cubic splines of tree size were not significant predictors of BAI in all three species (*P* > 0.10).

## Discussion

The 18‐yr experimental rainfall reduction did not decrease tree fecundity, contrary to predictions. The year‐to‐year variability and synchrony of reproduction were also unaffected by the levels of drought induced by our experiment. Drought, however, affected the allocation of resources in *Q. ilex* and *A. unedo* but not the more drought‐tolerant *P. latifolia*. Production of larger numbers of fruits by both *Q. ilex* and *A. unedo* was associated with a stronger decrease in growth in the rainfall‐reduction plots compared with the control plots, suggesting that these species were able to maintain their fecundity by shifting their allocation of resources away from growth.

Theory predicts that tree reproduction will be sensitive to climate change, due to strong correlations between seed production and annual variation in the weather (Pearse *et al.*, 2016). Fruit production by the three species studied here was previously correlated with drought severity (Ogaya & Peñuelas, 2007a; Espelta *et al.*, 2008; Bogdziewicz *et al.*, 2017b), but fecundity was resistant to the level of drought induced by this experiment. The synchrony and interannual variability of reproduction were consequently also unaffected. More frequent adverse weather, such as drought, should increase variability and synchrony among years by decreasing reproduction in some years, thus strengthening the reinforcing effects of stored resources on the synchrony of reproductive variation among trees (Rees *et al.*, 2002; Espelta *et al.*, 2008; Bogdziewicz *et al.*, 2018, 2019; Wion *et al.*, 2019). Such effect is, however, only expected in the case of very intense drought episodes when reproductive failures happen more frequently (Espelta *et al.*, 2008), which was not the case in our forest. One possibility is that water stress induced by natural drought also includes decrease in atmospheric water availability. Our rainfall‐reduction treatment can only influence soil‐water availability. Thus, we are unable to isolate the effects of evaporative demand on plant stress, despite the fact that both soil moisture and evaporative demand independently limit and affect vegetation productivity and water use during periods of hydrologic stress (Breshears *et al.*, 2013; Novick *et al.*, 2016). This appears to be a promising avenue for future research.

Our results implied that the maintenance of fecundity under drought stress was possible by decreasing growth in *Q. ilex* and *A. unedo*, indicated by changes in the trade‐off between growth and reproduction with and without stress. The slope of the positive relationship between fruit production with growth was reduced by over 30% in *Q. ilex*, and 60% in *A. unedo* in drought treatment compared with the control. *P. latifolia* was in turn able to sustain both growth and reproduction under induced drought. Stem growth in the drought treatment was > 60% lower for *A. unedo*, > 17% lower for *Q. ilex*, and was unaffected for *P. latifolia* compared with the control plots (Barbeta *et al.*, 2013). Drought reduces transpiration by stomatal closure in *Q. ilex* and *A. unedo*, which decreases the assimilation of carbon (Limousin *et al.*, 2009; Ripullone *et al.*, 2009). Drought also increases litterfall, likely to be due to xylem cavitation that accelerates foliar senescence (Choat *et al.*, 2012; Liu *et al.*, 2015). Defoliation in turn decreases the carbon content of plant tissues (Rosas *et al.*, 2013), suggesting that drought stress decreased resource availability in *Q. ilex* and *A. unedo* and forced the trees to partition the limited resources to reproduction at the expense of growth, providing experimental evidence for intraspecific variability and phenotypic plasticity in the cost of reproduction due to habitat differences. Alternatively, the positive association between growth and fruit production could follow from both growth and reproduction responding similarly to water availability. If the drought treatment decreases variability in water availability, it could weaken the correlation between growth and fruiting. However, our data suggest that the variance in soil moisture was similar in both treatments (Levene’s test, *F* = 0.44, *P* = 0.51; Fig. 1d). Another important implication of these findings is that *Q. ilex* has avoided reduced growth associated with reproduction throughout most of its range (Pérez‐Ramos *et al.*, 2010; Fernández‐Martínez *et al.*, 2015), but our results imply that it may not continue do so in the near future due to the progressive increase in drought frequency predicted by models of global change.

Generally positive relationships between growth and reproduction in all three species, indicated that favourable meteorological conditions could increase the accumulation of resources and their subsequent allocation to both growth and reproduction in certain years (Norton & Kelly, 1988; Fernández‐Martínez *et al.*, 2015; Vergotti *et al.*, 2019). Nonetheless, the maintenance of reproduction at the expense of growth, together with the previously established link between drought, reduced growth, and elevated mortality at our site (Ogaya & Peñuelas, 2007b; Barbeta *et al.*, 2013; Liu *et al.*, 2015), supports well the theory of the cost of reproduction in plants, where current reproductive allocation at the expense of growth is predicted to influence the probability of future survival (Obeso, 2002). The lack of direct effects of drought on fecundity thus does not preclude indirect costs of fecundity from sustained lower growth rates, which may influence future reproduction.

The results of this study indicated substantial resistance of tree fecundity in a *Q. ilex* dominated forest subjected to an average 15% (median 13%) decrease in the amount of soil moisture. Decreased growth and aboveground net primary production, increased mortality, or reduced photosynthesis observed at the drought plots indicate that lack of the effect on reproduction is not a consequence of lack of stress induced by the experiment (Barbeta *et al.*, 2013; Ogaya *et al.*, 2014, 2019; Liu *et al.*, 2015). The length of the study provides consistency to these results. Growing evidence indicates that *Q. ilex* dominated forests are resistant to an increase in drought to some extent, suggesting that these ecosystems may adapt to a progressive increase in arid conditions (Peñuelas *et al.*, 2018). Our study, however, comes with an important warning. The species‐specific reductions in growth and increased mortality (Ogaya & Peñuelas, 2007b; Barbeta *et al.*, 2013) may indirectly affect future fecundity and ultimately shift community composition, even without immediate direct effects of drought on tree fecundity.

## Author contributions

All authors conceived the study, RO, JME and JP collected data, MB and MFM ran the analysis, all authors participated in the evaluation of the results, MB drafted the manuscript and all authors participated in the editing and approved the final version.
